# Scaffold Implant Into the Epididymal Adipose Tissue Protects Mice From High Fat Diet Induced Ectopic Lipid Accumulation and Hyperinsulinemia

**DOI:** 10.3389/fbioe.2020.00562

**Published:** 2020-06-16

**Authors:** Michael A. Hendley, Christopher Isely, Kendall P. Murphy, Hayley E. Hall, Prakasam Annamalai, R. Michael Gower

**Affiliations:** ^1^Biomedical Engineering Program, University of South Carolina, Columbia, SC, United States; ^2^Department of Chemical Engineering, University of South Carolina, Columbia, SC, United States

**Keywords:** diabetes, insulin resistance, adipose tissue, scaffold, resveratrol, bioengineering, tissue engineering

## Abstract

Ectopic lipid accumulation, the deposition of lipids in lean tissue, is linked to type 2 diabetes through an association with insulin resistance. It occurs when adipose tissue fails to meet lipid storage needs and there is lipid spillover into tissues not equipped to store them. Ectopic lipid contributes to organ dysfunction because lipids can interfere with insulin signaling and other signaling pathways. Clinical studies indicate that decreasing ectopic lipids through diet and exercise is effective in treating type 2 diabetes; however, its prevalence continues to rise. We propose that strategies to improve lipid handling in the adipose tissue would be adjunctive to healthy lifestyle modification and may address difficulties in treating type 2 diabetes and other syndromes spurred by ectopic lipid. Herein, we investigate biomaterial implants as a means to increase lipid utilization in adipose tissue through the recruitment of highly metabolic cells. Poly(lactide-co-glycolide) scaffolds were implanted into the epididymal fat of mice fed a high fat diet that overwhelms the adipose tissue and promotes ectopic lipid accumulation. Over 5 weeks, mice with scaffolds gained less weight compared to mice without scaffolds and were protected from hyperinsulinemia. These effects correlated with a 53% decrease in triglyceride in the gastrocnemius and a 25% decrease in the liver. Scaffolds increased CPT1A protein levels in the epididymal fat and histology revealed high expression of CTP1A in the cells infiltrating the scaffold relative to the rest of the fat pad. In addition, lacing the scaffold with resveratrol increased CPT1A expression in the epididymal fat over scaffolds with no drug; however, this did not result in further decreases in weight gain or ectopic lipid. Mechanistically, we propose that the cellular activity caused by scaffold implant mitigates the lipid load imposed by the high fat diet and leads to a substantial decrease in lipid accumulation in the muscle and liver. In conclusion, this study establishes that a tissue engineering approach to modulate lipid utilization in the epididymal fat tissue can mitigate ectopic lipid accumulation in mice fed a high fat diet with positive effects on weight gain and whole-body insulin resistance.

## Introduction

Mounting evidence supports that lipid accumulation in non-adipose tissues, such as the liver and muscle, is a contributive factor in the progression of type 2 diabetes (Shulman, 2014). Lipid stored in non-adipose tissue is commonly referred to as ectopic lipid and this can occur in situations such as chronic overnutrition associated with overweightness and obesity, but also in disorders in which the body is unable to maintain adipose tissue (i.e., lipodystrophy Petersen et al., 2002). Ultimately, ectopic lipid results from a failure of the adipose tissue to adequately store lipid. Lipid storage in the adipose tissue is accomplished by adipocytes (Rutkowski et al., 2015). These cells convert excess energy in the form of fatty acids into triglycerides and store them in a specialized lipid droplet. When energy balance is in deficit, fatty acids are released from triglyceride and transported to tissues via the blood stream. However, the adipocyte's ability to store triglyceride is finite and chronic overnutrition can lead to the buildup of lipid metabolites that disrupt the adipocyte's energy storage program, which can manifest as a mismatch between rates of triglyceride biosynthesis and lipolysis (Jönsson et al., 2019). The end result is elevated flux of fatty acids from the adipose tissue, which are then taken up in peripheral tissues, such as the liver and muscle, and stored as triglyceride. However, myocytes and hepatocytes may also become dysregulated by lipid species generated during triglyceride metabolism because they interfere with insulin signaling (Petersen and Shulman, 2018). In the muscle, disrupted insulin signaling decreases glucose uptake. In the liver, the inability to respond to insulin maintains gluconeogenesis even though glucose is plentiful. Ultimately, lipid spillover from the adipose tissue to muscle and liver results in hyperglycemia that damages blood vessels and their associated organs. In order to compensate, the islets of Langerhans in the pancreas elevate insulin levels in the blood (i.e., hyperinsulinemia) to overcome insulin resistance. However, in some people, islets become stressed and die, insulin production decreases below critical levels, and type 2 diabetes ensues.

The ectopic lipid hypothesis is supported by clinical studies that indicate decreasing lipid load in the muscle and liver with diet and exercise (Snel et al., 2012), pharmacological intervention (Mayerson et al., 2002; Bajaj et al., 2003), or bariatric surgery (Lassailly et al., 2015; Angelini et al., 2019) mitigates hyperglycemia and hyperinsulinemia. However, the prevalence of diabetes continues to increase (Centers for Disease Control Prevention, 2017) suggesting that diet and exercise alone may not be sufficient to induce remission in most subjects. In addition, FDA approved drugs that promote improved lipid storage in the adipose tissue (i.e., the thiazolidinediones) are hindered by off-target effects such as cardiotoxicity (Davidson et al., 2018). Finally, bariatric surgery is highly invasive, permanently life-altering, and there is risk of complications such as infection and unwanted dysfunction of the digestive tract (Lim et al., 2018). Cumulatively, the available data suggests that what may be needed is a localized strategy to improve lipid handling in the adipose tissue that can be implemented in conjunction with efforts to restore energy balance through diet and exercise.

We propose that modulating lipid handling in the adipose tissue with biomaterials is a novel and promising strategy. Indeed, biomaterial implant instigates a chain reaction of cellular activity in the underlying tissue (Chung et al., 2017). Acutely, immune cells are recruited to survey for infection, promote clotting, and clear damaged cells and extracellular matrix. Long term, immune cells, in conjunction with fibroblasts, degrade the biomaterial or integrate it with the host tissue. Macrophages are a key immune cell type involved in this cascade and are well-known to fuse into giant cells in order to facilitate degradation of the biomaterial (Sheikh et al., 2015). To our knowledge, the metabolic activity associated with biomaterial implant has not been directly measured; however, we hypothesize that metabolic activity within the tissue following biomaterial implant would significantly increase, and it is possible that metabolic activity might be forever increased due to the novel microenvironment consisting of immune cells, giant cells, fibroblasts, and vasculature that is established in the wake of biomaterial integration (Chung et al., 2017). Interestingly, fatty acids are a key energy substrate that fuel macrophage activity (and the activity of other cells) during both inflammation and wound healing (Remmerie and Scott, 2018), and thus, are likely to be utilized during the immune response to biomaterial implant. Taken together, we hypothesized that biomaterial scaffolds that integrate with adipose tissue would mitigate lipid overflow from the adipose tissue into non-adipose tissues. We set out to test this hypothesis in high fat diet fed mice using poly(lactide-co-glycolide) (PLG) scaffolds whose implant results in immune cell infiltration into the adipose tissue (Gower et al., 2014), but does not result in increased expression of IL-6 and TNF-alpha (Murphy et al., 2018), prototypical cytokines whose expression correlate with adipose tissue dysfunction (Cao, 2014).

High fat diet feeding of mice is widely used to study lipid overload of the adipose tissue and ectopic lipid deposition. In particular, when male C57BL/6 mice are fed a high fat diet, their adipose tissues expand rapidly (especially the epididymal fat pad) but fail to store the excessive levels of dietary lipid. Triglyceride content in the liver and muscle increases within 1 and 3 weeks, respectively, and coincides with defects in insulin action in these tissues (Turner et al., 2013). Recently, we reported that PLG scaffolds (introduced above) decreased total body fat when implanted into the epididymal fat pad of mice fed a high fat diet (Hendley et al., 2019). We found that this effect coincided with the influx of macrophages and other non-adipocytes into the scaffold implant site as well as the formation of giant cells around pieces of the degrading scaffold. However, we did not establish if lipid accumulation in the liver and muscle correlated with the decrease in body fat, nor did we investigate changes in expression of molecular machinery at the implant site that might play a role in decreasing body fat. In the studies detailed herein, we test the hypothesis that implant of PLG scaffolds into the epididymal fat pad of high fat diet fed mice will decrease body fat gain and that these effects will coincide with (i) decreased ectopic lipid deposition in the liver and muscle and (ii) markers of improved insulin sensitivity. At the implant site, we investigate the impact of scaffolds on CPT1A levels, the enzyme that catalyzes the rate-limiting step in fatty acid catabolism (i.e., oxidation) and utilize histology to identify cell types that express this enzyme. Finally, we strengthen our conclusions by engineering the scaffold to release resveratrol, a small molecule established to enhance fatty acid oxidation via its modulation of CPT1A (Mercader et al., 2011; Imamura et al., 2017) and investigate the effects of its localized delivery to the epididymal fat on mice fed a high fat diet.

## Methods

### Particle Fabrication

Particles were produced by an oil-in-water emulsion-solvent evaporation technique. The oil phase consisted of a 3:1 mixture of dichloromethane (Sigma, St. Louis, MO) and ethanol (Sigma, St. Louis, MO) containing 6% w/v PLG (purchased from Evonik, Birmingham, AL, 75:25 mol ratio lactide to glycolide, 0.76 dL/g) and 10 mg/mL of trans-resveratrol (R5010, Sigma, St. Louis, MO). The aqueous phase consisted of 1% w/v poly(vinyl alcohol) (Sigma, St. Louis, MO) dissolved in ultrapure water. The oil phase was homogenized with the aqueous phase using a 1:7 volume ratio with a benchtop homogenizer (Kinematica, Bohemia, NY). The emulsion was then immediately added to ultrapure water and stirred for 5 h to allow particles to harden and organic solvents to evaporate. Particles were collected via centrifugation, washed with ultrapure water, and lyophilized.

### Scaffold Fabrication

Scaffolds were fabricated by mixing PLG particles with 250–500 μm NaCl particles (Sigma, St. Louis, MO) in a 1:30 ratio and the mixture was then pelleted in a die. Scaffolds were gas-foamed using 800 psi CO_2_ at room temperature in a custom-made pressure vessel. The salt porogen was removed by washing in ultrapure water. Complete salt removal was confirmed by scaffold weight and microscopy. Scaffolds that did not contain resveratrol were fabricated from particles produced using an oil phase that did not contain resveratrol or ethanol. From here on resveratrol loaded scaffolds are referred to as “resveratrol scaffolds” and scaffolds that do not contain resveratrol are referred to as “blank scaffolds.”

### Scanning Electron Microscopy of Scaffolds

Scanning electron microscopy was performed using a technique published previously (Hendley et al., 2019). Briefly, scaffolds were placed on aluminum SEM stubs coated with carbon adhesive tape. Scaffolds were then sputtered with gold for 1 min 3 times to ensure complete coating. Images were taken on a TESCAN Vega3 scanning electron microscope (Warrendale, PA) at 10 kV.

### Quantification of Resveratrol Within Scaffolds

Resveratrol scaffolds were dissolved in dimethyl sulfoxide (DMSO, Sigma, St. Louis, MO) and samples were loaded into a UV-Star® 96 well plate (Greiner Bio-One, Monroe, NC) and scanned for absorption at 330 nm using a Biotek Synergy 2 plate reader (Winooski, VT). Resveratrol content was determined with an 8-point standard curve produced by making ½ dilutions of a 1 mg/mL resveratrol solution in DMSO. Resveratrol used in the standard curve was the same product that was encapsulated into the scaffold (R5010, Sigma, St. Louis, MO). All resveratrol standards contained PLG at the same concentration as the scaffold samples.

### Animal Studies

All procedures were performed in accordance with NIH Guidelines for Care and Use of Animals and were approved by the Institutional Animal Care and Use Committee at the University of South Carolina under protocol number 2236-100943-121214. Male C57BL/6J mice were purchased from Jackson Laboratory at 6 weeks of age. All animals were housed with *ad libitum* access to water and food in a temperature and humidity controlled room (65–75°F and 40–60% humidity) with a 12-h light/12-h dark cycle. Mice were allowed to acclimate for 2 weeks prior to study. A total of 52 mice were used in the study: 20 mice in the 5-week study with 5 mice per group; 20 mice in the 2-week study with 5 mice per group; and 12 mice in the study that assessed resveratrol content in fat pads with 3 mice per group.

### High Fat Diet Feeding

After 2 weeks of acclimation, mice were placed on a 60% high-fat diet (D12492, Research Diets, New Brunswick, NJ) while a control group was kept on a normal diet (Teklad Diet 8904, Envigo, Indianapolis, IN) in order to demonstrate normal mouse growth and food intake. On a caloric basis, the high-fat diet consisted of 60% fat from lard, 20% carbohydrate, and 20% protein (total 5.24 kcal/g), whereas the normal diet contained 12% fat, 66% carbohydrate, and 22% protein (total 3.0 kcal/g). Mice were kept on the high fat diet for 1 week prior to scaffold implantation and remained on the diet for the remainder of the experiment (2 or 5 weeks).

### Scaffold Implantation

Mice received scaffold implants into the epididymal fat as previously described (Gower et al., 2014). Briefly, mice were anesthetized with a 2% mixture of isoflurane and oxygen (2 L/min), and the abdominal midline was shaved and prepped in a sterile fashion. Following a lower abdominal midline incision, each epididymal fat pad was wrapped around the scaffolds so that the scaffolds were in the center of the tissue. (Owing to the scaffold's hydrophobicity, light weight, and high surface area, it readily adheres to the fat pad and does not require sutures to stay in place). The abdominal wall was then closed with a running stitch, and the skin was closed with wound clips. All mice received two scaffolds per fat pad (i.e., each mouse received 4 scaffolds). In mice receiving resveratrol scaffolds, the resveratrol dose was calculated to be ~270 μg or 14 mg/kg. The amount of polymer delivered was ~6 mg for both groups of mice (i.e., resveratrol scaffolds and blank scaffolds). Additionally, a group of mice received the complete implant procedure but no scaffold, referred to as “sham” from this point forward.

### Body Weight Measurements and Dual Energy X-Ray Absorptiometry (DEXA)

Body composition was assessed using DEXA scans (Lunar PIXImus, GE Medical Systems Lunar, Madison, WI). Each mouse was weighed on a scale and then anesthetized for the duration of the DEXA procedure (5 min) by exposure to 2% isoflurane-oxygen gas via a nose cone. Mice were placed on the scanner bed in the prone position, with the limbs and tail stretched away from the body. One scan per mouse was performed and analyzed with PIXImus software (GE Medical Systems Lunar, Madison, WI). The head was excluded from calculation using a manual ROI. The PIXImus was calibrated with an aluminum/Lucite phantom (corresponding to bone mineral density = 0.0592 g/cm^2^ and 12.5% fat) on each day of testing according the manufacturer's instructions.

### Intraperitoneal Glucose Tolerance Test (IPGTT)

Mice were fasted for 6 h (8–2PM) before receiving injections of D-glucose (Sigma, St. Louis, MO) into the intraperitoneal cavity. Glucose administered was normalized to the lean mass calculated via a DEXA scan that was conducted 1-day prior (2g glucose per kg lean mass). Blood samples were collected via the tail vein at 0, 15, 30, 60, 90, and 120 min after glucose administration. Glucose measurements were obtained using a handheld glucose meter (Accu-Chek Nano, Roche Diabetes Care, Indianapolis, IN).

### Food Intake Measurements

Food was weighed at the beginning and end of each week and the total grams of food consumed was calculated. To obtain kcal per mouse, weight of food consumed was normalized by the number of mice in the cage and multiplied by the calorie density in the food (5.24 kcal/gram).

### Blood and Tissue Collection

5-week study (HFD for one week, scaffold implant on day 7, andHFD for five additional weeks): 5 weeks after scaffold implant, but 2 days before the IPGTT, the mice were fasted for 6 h (8AM−2PM). Blood (100 μL) was then collected retro-orbitally via microhematocrit capillary tubes (VWR, Radnor, PA) and transferred to EDTA coated BD microtainer tubes (VWR, Radnor, PA). (This blood was used to measure fasting insulin levels via ELISA). Then, the day after the IPGTT, the mice were fasted for 6 h and euthanized via cervical dislocation under isoflurane anesthesia. Both epididymal fat pads, both gastrocnemius muscles, and the entire liver were excised, washed in sterile saline, weighed, and frozen in tubes placed on dry ice. Tissues were then stored at −80°C until processing.

2-week study (HFD for one week, scaffold implant on day 7,and HFD for two additional weeks): 2 weeks after scaffold implant, mice were fasted for 6 h (8AM−2PM). The mice were then euthanized via cervical dislocation under isoflurane anesthesia. Both epididymal fat pads, both gastrocnemius muscles, and the liver were excised, washed in sterile saline, and weighed. Tissues were frozen on dry ice or fixed in in 4% formaldehyde solution in sterile PBS as needed.

### Insulin Enzyme Linked Immunosorbent Assay

Blood collected into EDTA coated tubes was centrifuged for 15 min at 10,000 g and 4°C and plasma was isolated. Insulin ELISA kit was purchased from ALPCO (80-INSMS-E01, Salem, NH). The assay was performed according to the manufacturer's instructions.

### Triglyceride Extraction and Quantification

Triglycerides were quantified using a colorimetric assay purchased from Cayman Chemical (Item No. 10010303, Ann Arbor, MI). Triglycerides were extracted from previously frozen tissues as specified by the manufacturer. Briefly, tissues (i.e., gastrocnemius muscle or liver) were suspended in NP40 buffer (Cayman Chemical, Ann Arbor, MI) supplemented with protease inhibitor cocktail (Thermo Scientific, Waltham, MA). Tissues were then minced using sterile forceps and homogenized using a benchtop homogenizer (Kinematica, Bohemia, NY). Homogenates were subject to centrifugation at 10,000 g at 4°C for 10 min and the entire supernatant was transferred to a fresh tube. Samples were loaded into a 96 well-plate and the assay was performed according to the manufacturer's instructions.

### Western Blotting

Whole tissues were homogenized in RIPA buffer (Thermo Scientific, Waltham, MA) supplemented with protease inhibitor cocktail (Thermo Scientific, Waltham, MA), phospho-stop (Roche, Indianapolis, IN), and phenylmethane sulfonyl fluoride (Sigma, St. Louis, MO). Following homogenization, protein was isolated via centrifugation at 18,000 g at 4°C for 45 min. Protein concentrations were determined using a BCA assay (Thermo Scientific, Waltham, MA). Lysates (30 ug adipose tissue) were separated by SDS-polyacrylamide gel electrophoresis (SDS-PAGE) using a 10% gel. Proteins were then transferred to a nitrocellulose membrane (GE Healthcare Life Sciences, Madison, WI) and incubated with appropriate antibodies. Secondary antibody was a horseradish peroxidase (HRP)-conjugated anti-rabbit immunoglobulin G (IgG) grown in goat (polyclonal, ab97051, Abcam, Cambridge, UK). Proteins were visualized using SuperSignal West Pico substrate (Thermo Scientific, Waltham, MA). Antibodies to total Akt (polyclonal, 9272), phospho-Akt ser 473 (polyclonal, 9271), and GAPDH (clone 14C10, 2118) were purchased from Cell Signaling Technology (Danvers, MA) and used at a 1:1,000 dilution. The antibody for Glut4 (polyclonal, G4048) was purchased from Sigma Aldrich (St. Louis, MO) and used at a 1:1,000 dilution. The antibody for CPT1A (clone number EPR21843-71-2F) was purchased from Abcam (Cambridge, UK) and used at a 1:1,000 dilution.

### Histological Staining and Immunohistochemistry

Adipose tissues were fixed in a 4% formaldehyde solution and embedded into paraffin. Five-micron sections were then serially cut with a Reichert-Jung 2030 rotary microtome (Leica Microsystems, Buffalo Grove, IL) and mounted onto slides. Sections were either stained with hematoxylin and eosin or probed for CPT1A using immunohistochemistry. For immunohistochemistry, antigen retrieval was performed using citrate buffer with a pH of 6.0. Slides were then blocked in a solution of 10% goat serum (Sigma, St. Louis, MO), 1% BSA (Millipore (Sigma, Burlington, MA) and TruStain FcX (Biolegend, San Diego, CA) at a concentration of 10 μg/mL for 1 h. The rabbit anti-CPT1A antibody that reacts with mouse (clone number EPR21843-71-2F) was purchased from Abcam (Cambridge, UK). CPT1A antibody binding was visualized using an HRP labeled polymer that is conjugated to goat anti-rabbit immunoglobulins (EnVision+/HRP, Rabbit) and chromogen solution (liquid DAB+) by following the manufacturer's instructions (Agilent Dako, Santa Clara, CA).

To confirm specific CPT1A antibody binding, several controls were carried out. First, we determined that probing epididymal fat tissue sections (with and without scaffolds) with the EnVision+ polymer followed by DAB+ did not yield a signal above background. This indicated that the EnVision+ reagent did not bind to the adipose tissue or the scaffold microenvironment. Second, we determined that probing the epididymal fat tissue sections (with and without scaffolds) with a polyclonal rabbit antibody with no known specificity (ab37415, Abcam, Cambridge, UK) followed by EnVision+ and then DAB+ did not yield a signal above background. This suggested that non-specific binding of the CPT1A antibody was not the source of signal in our histological analyses.

### Measuring Resveratrol in Epididymal Fat Pads

Resveratrol was extracted from epididymal fat pads based on a previously published protocol (Juan et al., 2010). Each fat pad was added to 2 mL of an acidified methanol solution consisting of 80% (v/v) methanol (Sigma, St Louis, MO), 17.5% ultrapure water (Sigma, St Louis, MO), 2.5% acetic acid (Sigma, St Louis, MO) and 10 μL of 15% (v/v) ascorbic acid (Sigma, St Louis, MO). Fad pads were minced with forceps and homogenized using a Polytron tissue homogenizer (Kinematica, Bohemia, NY). The homogenizer was cleaned twice with 1 mL of the acidified methanol solution resulting in a final volume of 4 mL per tissue. Following homogenization, samples were vortexed for 5 min and centrifuged at 3,000 g for 30 min at 4°C. The supernatant was transferred to a clean tube and the solid residue was treated 2 more times with the procedure outlined above. The supernatant was then reduced to a final volume of ~1 mL with a rotary evaporator, passed through a 3 kDa molecular weight cutoff filter, and stored in an amber vial at −20°C prior to HPLC analysis.

A Thermo Ultimate 3,000 binary gradient system (Thermo Scientific, Waltham, MA) was used for HPLC analysis. The column used was a Chromegabond WR C18 (ES Industries, West Berlin, NJ) and the flow rate was kept at 0.2 mL/min. The UV detector was Agilent G1315B Diode Array Detector (Agilent Technologies, Santa Clara, CA) set for 306 nm. Solvent A was water and Solvent B was Acetonitrile (Sigma, St Louis, MO). From 0 and 30 min solvent was 90% A and 10% B. From 30 to 31 min solvent was 5% A and 95% B. From 31 min on solvent was 90% A and 10% B. HPLC/UV-Vis was used to detect resveratrol. A stock of trans-resveratrol (R5010, Sigma, St. Louis, MO) was used to determine the correct elution time, which was 12 min.

### Statistical Analysis

Data is presented as mean ± standard error of the mean. All statistical analyses were conducted using GraphPad Prism software version 8 (GraphPad Software, San Diego, CA). Data sets comparing three or more groups were analyzed using a one-way ANOVA with Tukey's multiple comparisons test. Data sets comparing two or more groups over time were analyzed using a two-way ANOVA with Tukey's multiple comparisons test. P values less than 0.05 were considered significant.

## Results

### Characterization of Scaffolds

**Figure 1:**
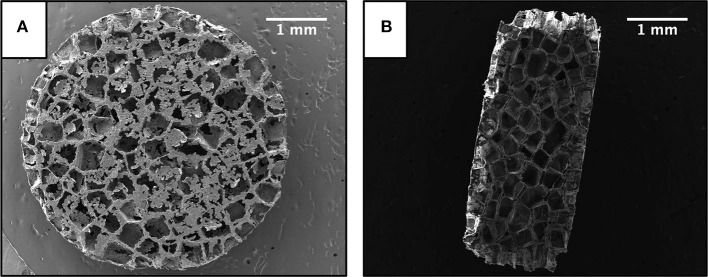
Scanning electron microscope images of a scaffold. **(A)** Top profile. **(B)** Cross sectional profile. Scale bar is 1 mm.

Representative SEM images of a scaffold are presented in Figure 1. Scaffold size and structure was unaffected by encapsulating resveratrol into the PLG particles used to form the scaffolds, which is consistent with a previous study (Murphy et al., 2018). Scaffolds were 5 mm in diameter and 2 mm in height with an average pore size of 370 ± 58 μm. Drug loading was 45 ± 3 μg of resveratrol per mg of polymer. Blank and resveratrol scaffolds had similar weights (Table 1); thus, the only difference detected between the two types of scaffolds was resveratrol content.

**Table 1:** Resveratrol loading and scaffold weights.

	**Resveratrol loading (μg/mg)**	**Scaffold weight (mg)**
Blank	N/A	1.55 ± 0.07
Resveratrol	45 ± 3	1.52 ± 0.02

### Impact of Scaffolds on Mice Challenged With a High Fat Diet

**Figure 2:**
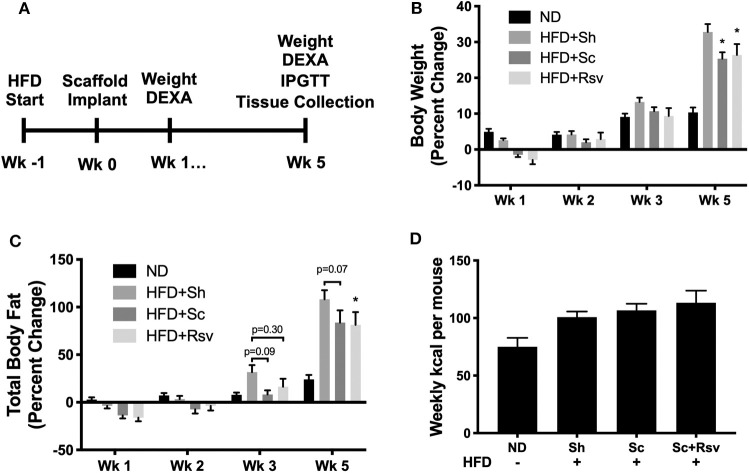
Effect of scaffolds on body composition in high fat diet fed mice. **(A)** Timeline of the study. **(B)** Percent change in body weight relative to the day of scaffold implant. Normal diet (ND) mice did not receive the implant procedure and demonstrate growth kinetics of unmanipulated mice on a chow diet. **(C)** Percent change in fat mass relative to the day of scaffold implant. **(D)** Weekly kcal intake per mouse over the 5-week experiment. For body weight and composition, statistics were calculated by two-way ANOVA with a Tukey's multiple comparisons test. For food consumption, statistics were calculated by one-way ANOVA with a Tukey's multiple comparisons test. *p < 0.05 vs. HFD+Sh. For all conditions and timepoints *n* = 5 mice. ND, normal diet; HFD, high fat diet; Sh, sham; Sc, blank scaffold; Sc+Rsv, resveratrol scaffold.

The experimental timeline for the study is depicted in Figure 2A. After 1 week of high fat diet feeding, which increases body fat and establishes insulin resistance (Turner et al., 2013), we implanted the scaffolds and then continued the mice on the diet for five additional weeks. Each mouse received two scaffolds per fat pad (~6 mg of PLG). Based on loading measurements depicted in Table 1, the resveratrol dose was ~270 μg or 14 mg/kg. Five weeks after scaffold implant, mice with scaffolds, regardless of resveratrol content, gained significantly less weight compared to sham mice (Figure 2B). Body composition analysis using DEXA indicated that mice with resveratrol scaffolds gained significantly less body fat compared to sham mice. The mice with blank scaffolds showed trends of lower body fat compared to sham, but statistical significance (*p* < 0.05) was not achieved (Figure 2C); however, there was not a significant difference between the two scaffold groups. Scaffold implant did not impact gains in lean mass (data not shown). These effects occurred without changes in food consumption between the high fat diet fed groups (Figure 2D). Taken together, the data indicate that scaffold implant results in a decrease in body weight gain caused by the high fat diet and suggest this is achieved through means other than decreasing food intake. Furthermore, the data suggest the scaffold, and not the incorporation of resveratrol, is responsible for these effects.

**Figure 3:**
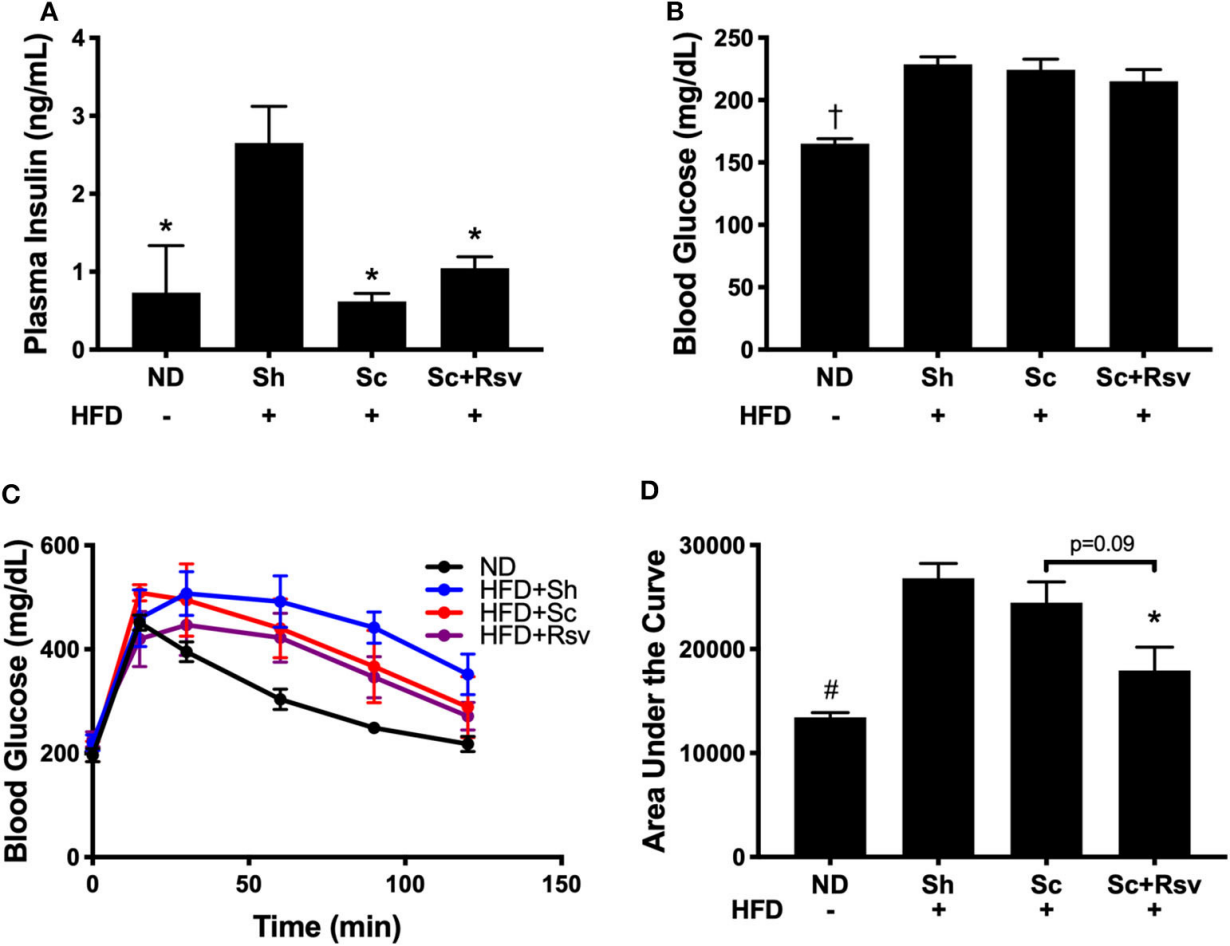
Fasting insulin and glucose levels and glucose tolerance 5 weeks after scaffold delivery in mice fed a high fat diet. **(A)** Fasting insulin measured in plasma by ELISA. **(B)** Fasting glucose measured in whole blood by glucometer. **(C)** Blood glucose vs. time during an intraperitoneal glucose tolerance test and **(D)** area under the curve. Statistics were calculated by one-way ANOVA with a Tukey's multiple comparisons test. *p < 0.05 vs. HFD+Sh. ^†^*p* < 0.05 vs. HFD+Sh, HFD+Sc, and HFD+Rsv. ^#^*p* < 0.05 vs. HFD+Sh and HFD+Sc. *n* = 5 mice per group.

In this mouse model, increased body fat correlates with whole body insulin resistance that manifests as hyperinsulinemia (i.e., elevated insulin levels in the blood), while reductions in body fat, correlate with improved insulin sensitivity (Lagouge et al., 2006). Indeed, mice fed the high fat diet, but did not receive scaffolds (sham), had significantly higher fasting plasma insulin levels compared to mice fed a normal diet (Figure 3A). In contrast, mice that received scaffolds, regardless of resveratrol content, had significantly lower fasting insulin levels relative to sham mice (Figure 3A). In addition, both scaffold groups had similar fasting glucose levels (Figure 3B). Taken together, high fat diet induced hyperinsulinemia was improved in mice with scaffolds (irrespective of drug) compared to sham mice. Interestingly, an intraperitoneal glucose tolerance test revealed that the resveratrol scaffold group was better able to clear a bolus of glucose from the blood stream compared to the sham group and there was a trend for improvement compared to the blank scaffold group (Figures 3C,D).

### The Effect of Scaffolds on Epididymal Fat Pad Weight and Triglyceride Accumulation in the Muscle and Liver

**Figure 4:**
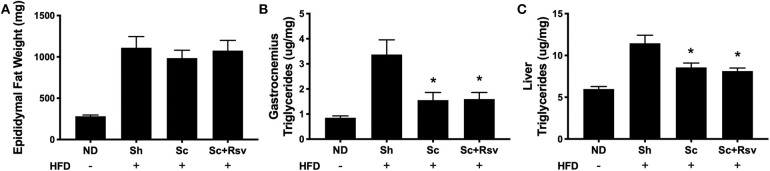
The effect of scaffolds on epididymal fat pad weight and triglyceride accumulation in the muscle and liver. Epididymal fat pad weight **(A)** and triglyceride content in the gastrocnemius muscle **(B)** and liver **(C)**. Statistics were calculated by one-way ANOVA with a Tukey's multiple comparisons test. * Represents *p* < 0.05 compared to HFD+Sh. *n* = 4-5 mice per group.

The decrease in body weight that accompanied scaffold delivery led us to investigate if the weight of the epididymal fat pad (the scaffold implant site) had decreased. While the high fat diet increased epididymal fat pad by 3-fold compared to normal diet fed mice, scaffold delivery did not decrease fat pad weight compared to sham controls (Figure 4A). We next investigated the triglyceride levels in the gastrocnemius and liver, as these are common sites of body fat accumulation during high fat diet feeding (Williams et al., 2014; Montgomery et al., 2017). Interestingly, mice treated with scaffolds, irrespective of drug loading, exhibited a 53% decrease in triglycerides in the gastrocnemius and 25% decrease in the liver compared with the sham group (Figures 4B,C). Taken together, the data suggests that the decrease in body weight observed with scaffold implant is due in part to decreased triglyceride accumulation in lean tissues including skeletal muscle and liver.

### Impact of Scaffolds on Proteins Associated With Insulin Signaling and Glucose Uptake in the Epididymal Fat Pad, Gastrocnemius, and Liver

**Figure 5:**
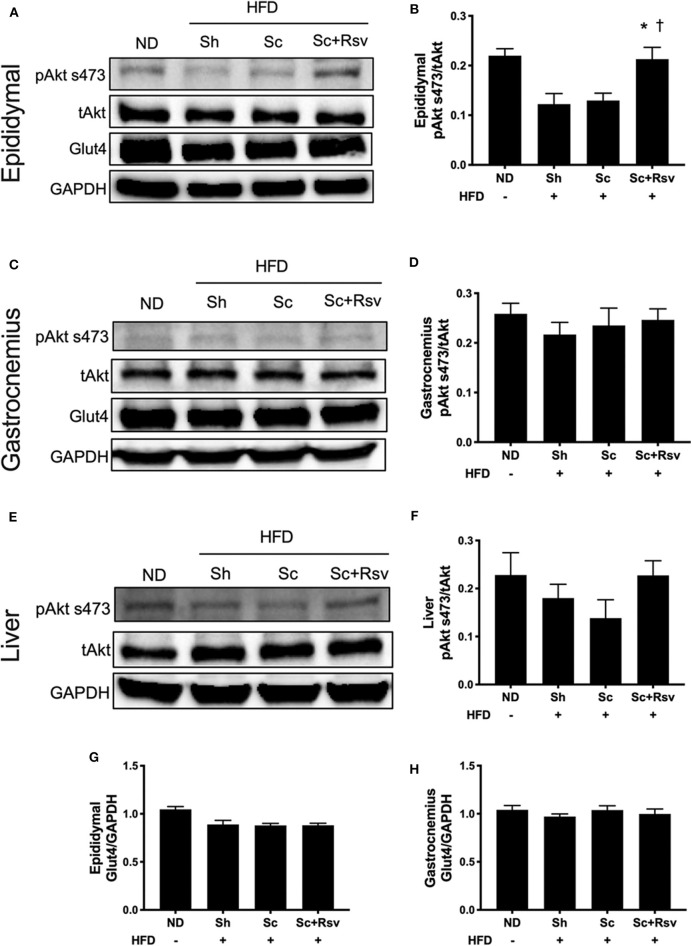
Impact of scaffolds on proteins associated with insulin signaling and glucose uptake in the epididymal fat pad, gastrocnemius muscle, and liver. **(A,C,E)** Representative western blots and **(B,D,F)** band quantification of Akt ratio for the epididymal fat pad **(A,B)**, gastrocnemius **(C,D)** and liver **(E,F)**. **(G,H)** Glut4 expression normalized to GPADH is depicted for the epididymal fat pad **(G)** and the gastrocnemius **(H)**. Statistics were calculated by one-way ANOVA with Tukey's multiple comparisons test. * Represents *p* < 0.05 compared to HFD+Sh, ^†^represents *p* < 0.05 compared to HFD+Sc. *n* = 5 mice per group.

Intact insulin signaling in peripheral tissues plays a large role in the regulation of blood glucose levels and Akt phosphorylation at serine 473 (pAkt s473) is the most commonly used readout of cellular insulin signaling (Petersen and Shulman, 2018). While all mice in the high fat diet groups (i.e., sham, blank scaffold, resveratrol scaffold) exhibited mild hyperglycemia (i.e., glucose abundance), only the sham group exhibited increased insulin levels, and we were curious if markers of insulin signaling might be different in the tissue of the sham mice compared to the scaffold treated mice. Interestingly, we found that relative levels of pAkt s473 (i.e., levels normalized to total Akt levels) were increased in the epididymal fat of the resveratrol group when compared to both the scaffold group and the sham group (Figures 5A,B). In addition, when we compared the Akt ratio of the resveratrol group with mice fed a normal diet (ND), we found that the ratios were similar. High fat diet feeding is established to decrease Akt phosphorylation at serine 473 in fat tissue (Hong et al., 2014; Ding et al., 2018) and our data is consistent with this (Figure 5B). In contrast, we did not detect differences in Akt ratio among the treatment groups in either the gastrocnemius (Figures 5C,D) or the liver (Figures 5E,F). The data suggest that resveratrol scaffolds may preserve insulin signaling in the epididymal fat.

Glut4 is the primary glucose transporter in the epididymal fat and the skeletal muscle and it is possible that the scaffolds might modulate expression of this protein. However, we did not detect an effect of blank scaffolds or resveratrol scaffolds on Glut4 in the epididymal fat (Figure 5G). In addition, we did not detect changes in Glut4 levels in the gastrocnemius (Figure 5H).

### The Effect of Scaffolds on CPT1A Protein Levels in Epididymal Fat

**Figure 6:**
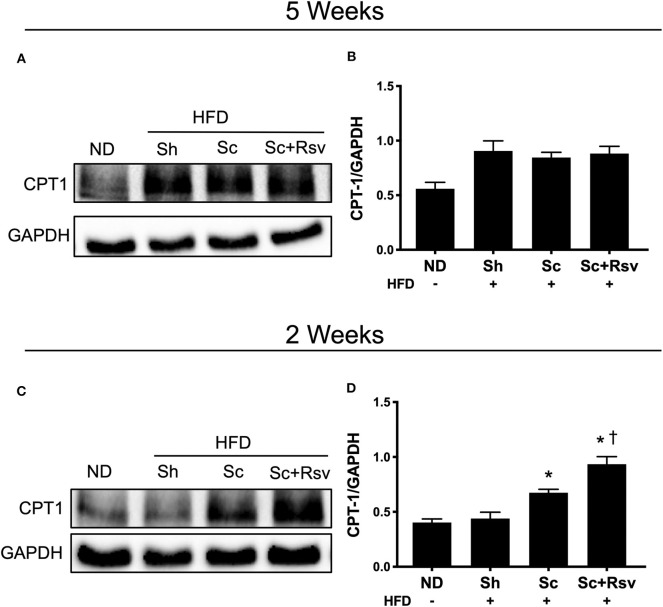
Effect of scaffolds on CPT1A in the epididymal fat. Representative western blots and band quantification for carnitine palmitoyltransferase 1 (CPT1A) in the epididymal fat **(A,B)** 5 weeks and **(C,D)** 2 weeks after implant. Statistics were calculated by one-way ANOVA with a Tukey's multiple comparisons test. * Represents *p* < 0.05 compared to HFD+Sh, ^†^ represents *p* < 0.05 compared to HFD+Sc. *n* = 5 mice per group.

CPT1 is the rate limiting enzyme in fatty acid oxidation (Mir et al., 2012). It is highly expressed in non-adipocytes relative to adipocytes (Malandrino et al., 2015). Having established previously that scaffold implant results in a microenvironment characterized by infiltration of several types of non-adipocytes including immune cells, endothelial cells and fibroblasts, we investigated if scaffold implant increased CPT1 expression. Specifically, we investigated expression of CPT1A, which is the dominant isoform expressed in all mammalian cells except muscle and brown fat (Mir et al., 2012). To begin with, we measured CPT1A in the same tissues assayed for Akt and Glut4; however, no differences were detected among the high fat diet fed groups (Figures 6A,B). We considered that the 5-week time point might be too late to see effects of the scaffolds on CPT1A; therefore, in a separate cohort of animals, following the exact experimental design depicted in Figure 2A, we conducted CPT1A measurements 2 weeks after scaffold implant. In this experiment, CPT1A was significantly higher in the epididymal fat of the blank scaffold group compared to the sham control (Figures 6C,D). Additionally, resveratrol scaffolds significantly increased CPT1A compared to both the blank scaffold group and the sham controls (Figure 6D).

### Histological Analysis of CPT1A Expression in the Epididymal Fat

**Figure 7:**
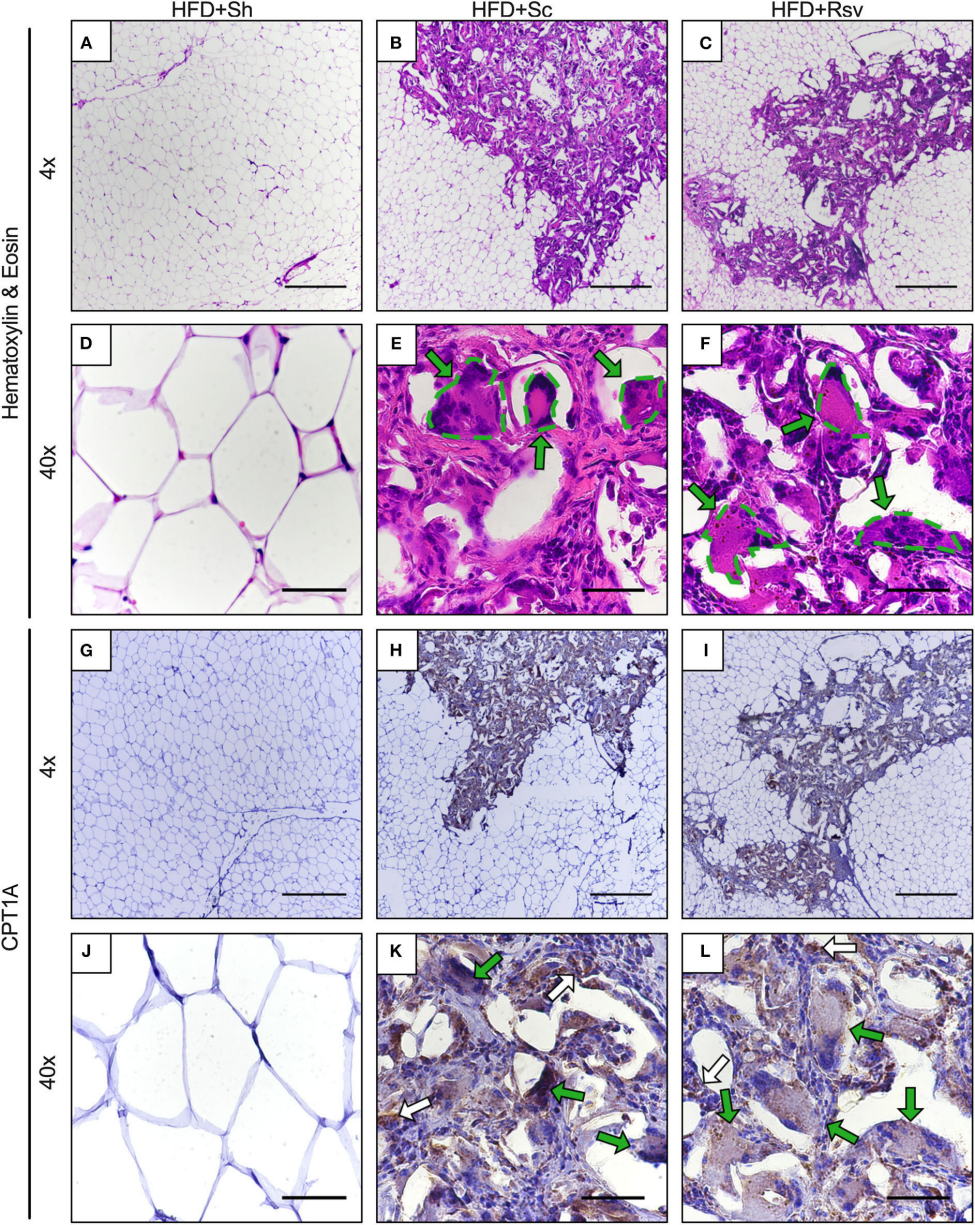
Cellular infiltration and CPT1A expression within epididymal fat pads 2 weeks after scaffold implant. Representative images of epididymal fat sections from high fat diet fed mice receiving sham procedure, blank scaffolds, or resveratrol scaffolds. Sections are from 2 weeks after scaffold implant. **(A–F)** Hematoxylin and eosin staining. **(E,F)** Dotted lines encircle multinucleated giant cells. **(G–L)** Immunohistochemistry for CPT1A. **(K,L)** Green arrows indicate multinucleated giant cells expressing CPT1A. White arrows indicate non-fused cells expressing CPT1A. Scale bars represent 500 μm for 4x images and 50 μm for 40x images. Data is a representative of 3 mice per condition and 3 separate sections per mouse.

To determine how scaffold implant might lead to increased CPT1A expression, tissue sections from the 2-week time point were evaluated using hematoxylin and eosin staining. Cellular infiltration was observed in the epididymal fat with blank and resveratrol scaffolds, which was not observed in the fat pads of the sham group (Figures 7A–C). Inspection at higher magnification showed similar levels of cell infiltration in both scaffold groups, with fibroblasts (oval nuclei) and mononuclear immune cells (circular nuclei) visible within the extracellular matrix deposited around the scaffolds. Additionally, multinucleated giant cells were seen surrounding pieces of the scaffold, which are irregular shaped white voids within the sections. To aid the reader, the giant cells are delineated with green dashes and arrows (Figures 7D–F). Immunohistochemistry indicated that CPT1A was expressed in cells within the scaffold implant site at much higher levels than anywhere else in the epididymal adipose tissue (Figures 7G–I). It was also observed that multinucleated giant cells expressed high levels of CPT1A (green arrows). There were also non-fused cells that also expressed CPT1A (white arrows). Further inspection of the implant site did not reveal any noticeable differences between the cell types expressing CPT1A in the blank vs. resveratrol scaffolds (Figures 7J–L).

### Resveratrol Content in Epididymal Fat Two and Five Weeks After Scaffold Implant

**Figure 8:**
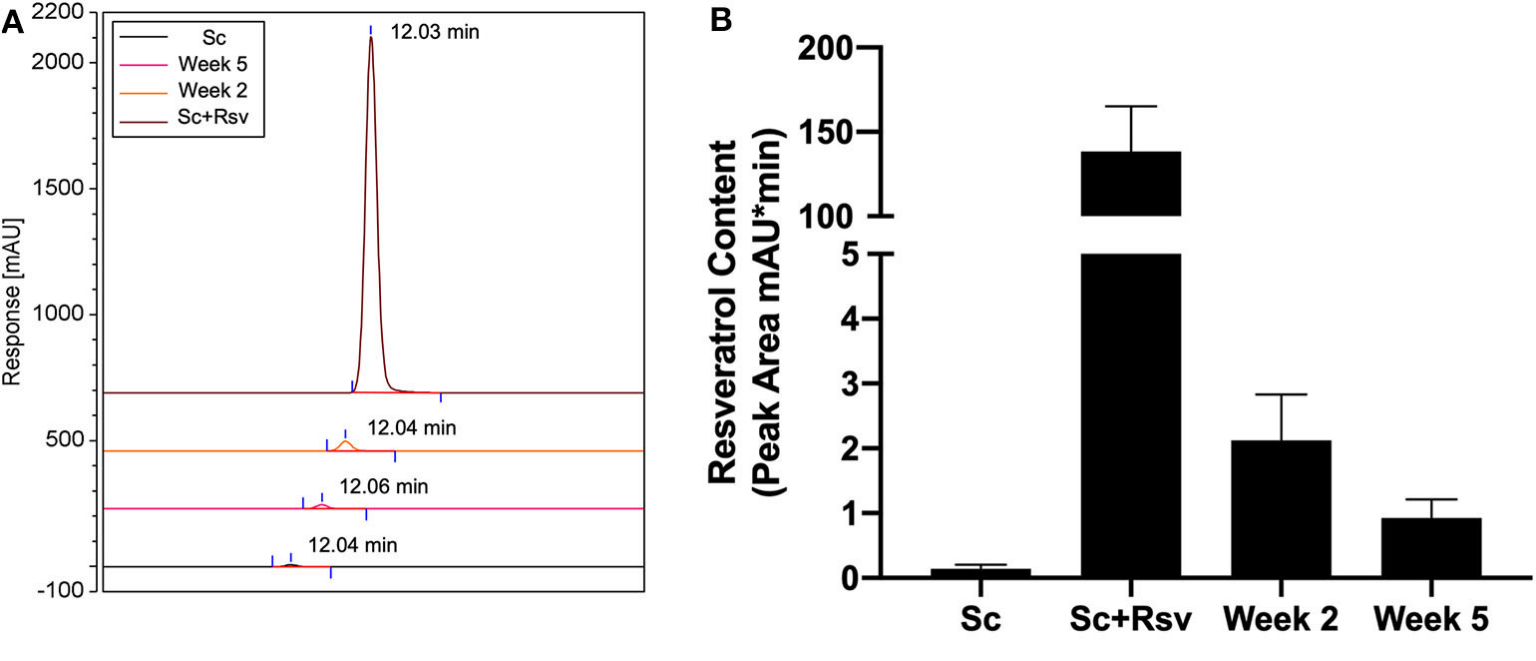
Resveratrol content in epididymal fat 2 and 5 weeks after scaffold implant. **(A)** HPLC chromatograms depicting resveratrol content in epididymal fat 2 and 5 weeks after implanting resveratrol scaffolds. For comparison, epididymal fat pads that did not receive scaffolds but were homogenized with either fresh resveratrol scaffolds (Sc+Rsv) or blank scaffolds (Sc) were included for comparison. The elution for each condition was offset by 10% when combined into a single graph. Elution times for resveratrol are labeled on the graph and were the same for all samples (~12 min). **(B)** Resveratrol content in the epididymal fat depicted as peak area calculated from HPLC chromatograms. *n* = 3 mice per group.

While an effect of resveratrol delivery on the glucose tolerance test was apparent 5 weeks after scaffold implant, it was unclear if resveratrol was present in the epididymal fat at that time point. To address this question, we measured resveratrol in epididymal fat pads 2 and 5 weeks after scaffold implant using HPLC (Week 2 and Week 5, respectively, Figure 8). For comparison, we also analyzed epididymal fat pads that did not receive scaffolds *in vivo* but were processed with either two fresh resveratrol scaffolds (Sc+Rsv, Figure 8) or two fresh blank scaffolds (Sc, Figure 8). Two scaffolds were used because in the *in vivo* study mice received two scaffolds per fat pad. HPLC indicated that resveratrol was present within the epididymal fat at both 2 and 5 weeks after scaffold implant, with ~2% of the initial resveratrol dose remaining at 2 weeks and ~1% remaining at 5 weeks (Figure 8).

## Discussion

The accumulation of unwanted lipids in peripheral tissues is linked to the development of insulin resistance that underlies type 2 diabetes. With limited treatment options and various drawbacks associated with current treatments, new strategies to mitigate this condition are needed. In this report we investigate a tissue engineering approach to mitigate ectopic lipids through implanting biomaterial scaffolds into the adipose tissue. Implant of scaffolds significantly decreased total body weight, which can likely be attributed to a decreased in total body fat, in mice fed a high fat diet. While we did not have sufficient power to detect significance between blank scaffolds and sham, the data suggest the scaffold, and not the incorporation of resveratrol, is most responsible for the observed effects. However, the incorporation of resveratrol did modestly improve the decrease in total body fat achieving statistical difference from sham. Interestingly, there was no change in epididymal fat pad weight suggesting that lipid accumulation was decreased elsewhere. This was confirmed upon investigation of triglyceride content in the peripheral tissues of the mice, which revealed a 53% reduction in triglyceride content in the skeletal muscle and a 25% reduction of triglycerides in the liver. Our results are on par with interventions such as exercise training which has been shown to decrease muscle triglycerides by ~30% (Ko et al., 2018) and pharmacological inhibitors of lipolysis which reduced muscle triglycerides by 53% (Schweiger et al., 2017). To our knowledge our results are the first to demonstrate that ectopic lipid deposition can be mitigated though the implant of polymer scaffold into the adipose tissue and these results are particularly promising as they were achieved with treatment by an FDA approved biomaterial. Additionally, a decrease in lipid content is also encouraging from an insulin sensitivity perspective as the buildup of ectopic lipids has been shown to disrupt insulin signaling in those tissues (Petersen and Shulman, 2018).

We hypothesized that scaffolds could be mitigating ectopic lipids in the peripheral tissues by increasing lipid utilization at the implant site. Supporting this hypothesis, we found that scaffolds increased CPT1A in the epididymal fat 2 weeks after implant, which is the rate limiting enzyme in fatty acid oxidation (Rutkowski et al., 2015) and an accepted marker for increased fatty acid utilization (Bruce et al., 2009). This finding is particularly important as there are currently no approaches to elevate CPT1A levels in targeted tissues other than viral vector delivery. Scaffold implant could represent a clinically translatable method to elevate this important therapeutic target. Taken together, we propose that scaffolds increased lipid utilization in the epididymal fat, which led to decreased lipid accumulation in lean tissues and therefore lower body fat.

Implanting scaffolds into the epididymal fat also results in cellular infiltration in the immediate vicinity of the scaffold. Because CPT1A expression is preferentially expressed in the stromal cells of the adipose tissue relative to adipocytes (Malandrino et al., 2015), we hypothesized that cells recruited to the adipose tissue by scaffold implant could represent the source of elevated CPT1A expression. Our immunohistochemistry study supports this hypothesis as we observed high levels of CPT1A expression in cells within the scaffold implant site relative to the adipocytes outside the implant site. We have reported previously that many of these cells include macrophages (Gower et al., 2014; Liu et al., 2016; Murphy et al., 2018; Hendley et al., 2019) and others have established that CPT1A is expressed by macrophages (O'Neill et al., 2016). Furthermore, we also demonstrate that multinucleated giant cells present in the implant site express CPT1A. To our knowledge, we are the first to report that CPT1A is expressed in these cells; however, it is not surprising because, based on current paradigms in immunometabolism, long-lived tissue resident immune cells involved in wound healing (as opposed to pathogen clearance) rely heavily on fatty acid catabolism, and CPT1A in particular, for their energy requirements (Diskin and Pålsson-McDermott, 2018). It is unclear why CTP1A is no longer elevated (relative to sham) 5 weeks after scaffold implant. We propose that the response to the scaffold could be resolving at this time point and future studies should focus on extending the scaffold's ability to elevate CPT1A at later time points. Collectively, we hypothesize that the macrophages and multinucleated giant cells that accumulate in the adipose tissue in response to scaffold implant are responsible for elevated CPT1A levels and that their metabolism plays a major role in the decreased body fat accumulation during a high fat diet by elevating the net utilization of fatty acids within the epididymal adipose tissue, and thus, keeping them from accumulating in non-adipose tissue such as the muscle and liver.

Resveratrol scaffolds increased CPT1A in the epididymal fat relative to blank scaffolds at the 2-week time point; however, this did not affect ectopic lipid deposition at 5 weeks. As discussed above, histology indicated that multinucleated giant cells, macrophages, and other non-adipose cells expressed CPT1A; however, to our knowledge, the effects of resveratrol on CPT1A expression in immune cells is unknown. In contrast, it is established that resveratrol increases CPT1A expression in adipocytes (Mercader et al., 2011; Imamura et al., 2017). In adipocytes, CPT1A expression is controlled by PPARγ, which resveratrol and its metabolites can bind and activate (Calleri et al., 2014). Therefore, it is possible that resveratrol could be acting through adipocytes to elevate CPT1A to a greater degree than its blank scaffold counterparts even though this elevation was not observable by immunohistochemistry. However, since PPARγ is also a master regulator of macrophage function (Chawla, 2010), it is possible that resveratrol increases CPT1A in these cells.

Blank and resveratrol scaffold groups had fasting plasma insulin levels that were indistinguishable, which suggested the groups had similar levels of whole-body insulin sensitivity (Ahren, 2004). Despite this, only the resveratrol scaffold group outperformed the sham group in the IPGTT. One possible explanation is that resveratrol could be acting locally at the implant site to promote glucose uptake. Resveratrol has been well-documented to promote glucose uptake *in-vitro* in various cell lines, including insulin resistant adipocytes (Chen et al., 2018) and co-cultured macrophages and adipocytes (Shin et al., 2016). Our HPLC results suggest resveratrol still remains in the adipose tissue at the time of the IPGTT, and therefore, it is plausible that it could be acting on these cell types to increase glucose uptake in the tissue during the tolerance test.

The most notable effect of resveratrol scaffolds was a significant improvement in glucose clearance from the blood stream compared to sham mice during a glucose tolerance test. This improvement was quantified by area under the curve (AUC) and the AUC for the resveratrol group was 36% lower than the sham group (lower numbers indicate faster glucose clearance). In comparison, resveratrol delivered orally to rodents or primates on a high fat diet decreases the AUC by ~30% compared to high fat diet fed controls (Baur et al., 2006; Lagouge et al., 2006; Beaudoin et al., 2013; Jimenez-gomez et al., 2013; Ding et al., 2018). Thus, the effects of resveratrol on glucose tolerance appear comparable between oral and scaffold delivery. However, the amount of resveratrol delivered was much lower for scaffold delivery (one dose of 14 mg/kg) vs. oral delivery, which requires daily doses in the range of 20–400 mg/kg/day depending on if the study takes place in mice (Baur et al., 2006; Lagouge et al., 2006; Hong et al., 2014; Ding et al., 2018), rats (Macarulla et al., 2009; Alberdi et al., 2011; Beaudoin et al., 2013), rhesus monkeys (Jimenez-gomez et al., 2013) or humans (Brasnyó et al., 2011; Timmers et al., 2011; Crandall et al., 2012). Thus, while there was no effect of direct resveratrol delivery to the adipose tissue on ectopic lipid accumulation, it is interesting to consider that biomaterial-based delivery of this nutraceutical to fat may be a promising approach to control post-prandial blood glucose levels, which is an important therapeutic target for both diabetics and pre-diabetics.

Considering all the data, we propose the following mechanism for the scaffold's therapeutic effects on ectopic lipid accumulation in mice fed a high fat diet. Scaffolds elevate CPT1A expression in the epididymal fat through recruiting highly metabolic cells to the epididymal adipose tissue. This effect is more pronounced at early time points after scaffold delivery and is lost by 5 weeks. We hypothesize that elevated CPT1A increases fatty acid utilization locally thereby preventing fatty acid accumulation throughout the mouse and, therefore, decreases total body weight. If fatty acid utilization was increased at the scaffold, which was not confirmed, this could have led to the decreased lipid accumulation in the muscle and liver, which in turn may have led to improved whole body insulin resistance reflected in the decreased plasma insulin levels. Overall, scaffold implant into the epididymal fat exerts interesting effects on body composition and insulin resistance in mice fed a high fat diet. Lacing the scaffolds with resveratrol improves upon these effects by further elevating CPT1A expression in the adipose tissue 2 weeks after scaffold implant and improving glucose tolerance 5 weeks after scaffold implant.

HPLC indicated that resveratrol was still present in the epididymal fat at both 2 and 5 weeks after scaffold implant, with ~2% of the initial dose remaining at 2 weeks and ~1% remaining at 5 weeks. However, *in vitro* release profiles of these scaffolds carried out in water at 37°C we published previously (Murphy et al., 2018), indicate that 40% of the resveratrol remains within the scaffold at 2 weeks. Accelerated *in vivo* release profiles have been reported in a variety of drug delivery systems including PLG microspheres (Sako et al., 2002; Nabais et al., 2007; Zolnik and Burgess, 2008). The accelerated *in vivo* release rates have been attributed to reactive oxygen species, acids, and degradative enzymes released by macrophages and multinucleated giant cells in direct contact with the polymer matrix (Anderson et al., 2008). Overall this data highlights the disparity between *in-vitro* and *in-vivo* release profiles and offers caution when extrapolating *in vitro* release data to *in vivo* scenarios. Furthermore, we suspect that if resveratrol release from the scaffold were better controlled, its delivery could further decrease ectopic lipid accumulation in the liver and/or muscle.

One caveat of this preclinical study is the invasiveness of the implant procedure. The visceral adipose tissue is located within the abdominal cavity, and therefore, we opted to use a surgical procedure to implant the scaffold. However, we believe that it would be possible for scaffolds to be implanted laparoscopically in larger animal models and in humans. Indeed, laparoscopic surgery has improved greatly over the past few decades with procedures such as appendectomies, splenectomies, and colon surgeries currently performed using these methods (Himal, 2002). We believe it is possible that the implantation of a biomaterial scaffold into the visceral adipose tissue would be no more invasive than these operations. The visceral adipose tissue is an important fat depot to target for this therapy because it has lower lipid storage capacity than subcutaneous fat (Spalding et al., 2017) and thus requires less lipid accumulation to become dysregulated. On top of this, the secretion of inflammatory cytokines from visceral adipose tissue has been linked to systemic inflammation in obese humans (Fontana et al., 2007). For these reasons, we focused on visceral adipose tissue for these studies; however, studies are underway in subcutaneous fat.

### Conclusion

Implant of tissue engineering scaffolds into the epididymal fat pad protects high fat diet fed mice from weight gain, ectopic lipid deposition in the liver and muscle, and hyperinsulinemia, which is indicative of whole-body insulin resistance in this mouse model. The protection correlates with accumulation of non-adipocytes and elevated CPT1A expression in the epididymal fat at 2 weeks after scaffold implant. Elevated CPT1A levels is due to non-adipocytes that reside in the scaffold, including multinucleated giant cells. Lacing the scaffold with resveratrol improved performance during a glucose tolerance test 5 weeks after scaffold implant but did not improve ectopic lipid profiles or hyperinsulinemia relative to “blank” scaffolds. HPLC indicated that 1% of the resveratrol payload was present at the time of the IPGTT and we propose that its presence may improve glucose uptake rates in the fat pad following an intraperitoneal injection of glucose. To our knowledge, we are the first to report that scaffolds implanted into the epididymal fat results in decreased ectopic lipid accumulation in muscle and liver in high fat diet fed mice. Furthermore, the scaffold's effect on ectopic lipid are similar to lifestyle and pharmacological interventions that have been reported using the high fat diet model. While the current delivery method is invasive and the lower limits on scaffold size and the importance of biomaterial form factor are currently unknown, this work establishes that cell-biomaterial interactions may be leveraged to modulate adipose tissue metabolism that impacts ectopic lipid deposition. As ectopic lipid contributes to the progression of type 2 diabetes, and current interventions have failed to decrease its prevalence, further study is warranted to define the role of material type, dose, form factor and delivery routes in this novel therapeutic approach, which is expected to be adjunctive to diet and exercise.

## Data Availability Statement

The datasets generated for this study are available by requesting to the corresponding author.

## Ethics Statement

The animal study was reviewed and approved by Institutional Animal Care and Use Committee at the University of South Carolina.

## Author Contributions

RG conceived of the original research idea. RG, MH, and PA designed the experiments. MH, CI, KM, and HH performed experiments and analyzed data. RG and MH wrote the manuscript. All authors have read and approved the final manuscript.

## Conflict of Interest

The authors declare that the research was conducted in the absence of any commercial or financial relationships that could be construed as a potential conflict of interest.

## References

[B1] Ahren B. (2004). The High-Fat Diet–Fed Mouse. Diabetes 53, 215–219. 10.2337/diabetes.53.suppl_3.S215 15561913

[B2] Alberdi G. Rodríguez V. M. Miranda J. Macarulla M. T. Arias N. Andrés-Lacueva C.. (2011). Changes in white adipose tissue metabolism induced by resveratrol in rats. Nutr. Metab. (Lond). 8:29. 10.1186/1743-7075-8-29 21569266 PMC3101135

[B3] Anderson J. M. Rodriguez A. Chang D. T. (2008). Foreign body reaction to biomaterials. Semin. Immunol. 20, 86–100. 10.1016/j.smim.2007.11.004 18162407 PMC2327202

[B4] Angelini G. Gissey L. C. Del Corpo G. Giordano C. Cerbelli B. Severino A.. (2019). New insight into the mechanisms of ectopic fat deposition improvement after bariatric surgery. Sci. Rep. 9, 1–15. 10.1038/s41598-019-53702-4 31754142 PMC6872729

[B5] Bajaj M. Suraamornkul S. Pratipanawatr T. Hardies L. J. Pratipanawatr W. Glass L.. (2003). Pioglitazone reduces hepatic fat content and augments splanchnic glucose uptake in patients with type 2 diabetes. Diabetes 52, 1364–1370. 10.2337/diabetes.52.6.1364 12765945

[B6] Baur J. A. Pearson K. J. Price N. L. Jamieson H. A. Lerin C. Kalra A.. (2006). Resveratrol improves health and survival of mice on a high- calorie diet. Nature 444, 337–342. 10.1038/nature05354 17086191 PMC4990206

[B7] Beaudoin M.-S. Snook L. A. Arkell A. M. Simpson J. A. Holloway G. P. Wright D. C. (2013). Resveratrol supplementation improves white adipose tissue function in a depot-specific manner in Zucker diabetic fatty rats. Am. J. Physiol. Integr. Comp. Physiol. 305, R542–51. 10.1152/ajpregu.00200.2013 23824959

[B8] Brasnyó P. Molnár G. A. Mohás M. Markó L. Laczy B. Cseh J.. (2011). Resveratrol improves insulin sensitivity, reduces oxidative stress and activates the Akt pathway in type 2 diabetic patients. Br. J. Nutr. 106, 383–389. 10.1017/S0007114511000316 21385509

[B9] Bruce C. R. Hoy A. J. Turner N. Watt M. J. Allen T. L. Carpenter K.. (2009). Overexpression of carnitine palmitoyltransferase-1 in skeletal muscle is sufficient to enhance fatty acid oxidation and improve high-fat diet-induced insulin resistance. Diabetes 58, 550–558. 10.2337/db08-1078 19073774 PMC2646053

[B10] Calleri E. Pochetti G. Dossou K. S. S. Laghezza A. Montanari R. Capelli D.. (2014). Resveratrol and its metabolites bind to PPARs. Chembiochem 15, 1154–1160. 10.1002/cbic.201300754 24796862 PMC4104805

[B11] Cao H. (2014). Adipocytokines in obesity and metabolic disease. J. Endocrinol. 220, T47–59. 10.1530/JOE-13-0339 24403378 PMC3887367

[B12] Centers for Disease Control and Prevention (2017). National Diabetes Statistics Report: Estimates of Diabetes and Its Burden in the United States. (Atlanta, GA: Centers for Disease Control and Prevention).

[B13] Chawla A. (2010). Macrophage activation by PPAR. Circ Res. 106, 1559–1569. 10.1161/CIRCRESAHA.110.216523 20508200 PMC2897247

[B14] Chen S. Zhao Z. Ke L. Li Z. Li W. Zhang Z.. (2018). Resveratrol improves glucose uptake in insulin-resistant adipocytes via Sirt1. J. Nutr. Biochem. 55, 209–218. 10.1016/j.jnutbio.2018.02.007 29554499

[B15] Chung L. Maestas D. R. Housseau F. Elisseeff J. H. (2017). Key players in the immune response to biomaterial scaffolds for regenerative medicine. Adv. Drug Deliv. Rev. 114, 184–192. 10.1016/j.addr.2017.07.006 28712923

[B16] Crandall J. P. Oram V. Trandafirescu G. Reid M. Kishore P. Hawkins M.. (2012). Pilot study of resveratrol in older adults with impaired glucose tolerance. J. Gerontol. Ser. A Biol. Sci. Med. Sci. 67, 1307–1312. 10.1093/gerona/glr235 22219517 PMC3670158

[B17] Davidson M. A. Mattison D. R. Azoulay L. Krewski D. (2018). Thiazolidinedione drugs in the treatment of type 2 diabetes mellitus: past, present and future. Crit. Rev. Toxicol. 48, 52–108. 10.1080/10408444.2017.1351420 28816105

[B18] Ding S. Jiang J. Wang Z. Zhang G. Yin J. Wang X.. (2018). Resveratrol reduces the inflammatory response in adipose tissue and improves adipose insulin signaling in high-fat diet-fed mice. PeerJ 6:e5173. 10.7717/peerj.5173 29967759 PMC6027658

[B19] Diskin C. Pålsson-McDermott E. M. (2018). Metabolic modulation in macrophage effector function. Front. Immunol. 9:270. 10.3389/fimmu.2018.00270 29520272 PMC5827535

[B20] Fontana L. Eagon J. C. Trujillo M. E. Scherer P. E. Klein S. (2007). Visceral fat adipokine secretion is associated with systemic inflammation in obese humans. Diabetes 56, 1010–1013. 10.2337/db06-1656 17287468

[B21] Gower R. M. Boehler R. M. Azarin S. M. Ricci C. F. Leonard J. N. Shea L. D. (2014). Modulation of leukocyte infiltration and phenotype in microporous tissue engineering scaffolds via vector induced IL-10 expression. Biomaterials 35, 2024–2031. 10.1016/j.biomaterials.2013.11.036 24309498 PMC3932667

[B22] Hendley M. A. Murphy K. P. Isely C. Struckman H. L. Annamalai P. Gower R. M. (2019). The host response to poly(lactide-co-glycolide) scaffolds protects mice from diet induced obesity and glucose intolerance. Biomaterials 217:119281. 10.1016/j.biomaterials.2019.119281 31260882 PMC6635072

[B23] Himal H. S. (2002). Minimally invasive (laparoscopic) surgery. Surg. Endosc. Other Interv. Tech. 16, 1647–1652. 10.1007/s00464-001-8275-7 12098024

[B24] Hong H. J. Kang W. Kim D. G. Lee D. H. Lee Y. Han C. H. (2014). Effects of resveratrol on the insulin signaling pathway of obese mice. J. Vet. Sci. 15, 179–185. 10.4142/jvs.2014.15.2.179 24675832 PMC4087218

[B25] Imamura H. Nagayama D. Ishihara N. Tanaka S. Watanabe R. Watanabe Y.. (2017). Resveratrol attenuates triglyceride accumulation associated with upregulation of Sirt1 and lipoprotein lipase in 3T3-L1 adipocytes. Mol. Genet. Metab. Reports 12, 44–50. 10.1016/j.ymgmr.2017.05.003 28580300 PMC5448575

[B26] Jimenez-gomez Y. Mattison J. A. Pearson K. J. Martin,-A. Palacios H. H. Sossong A. M.. (2013). Resveratrol improves adipose insulin signaling and reduces the inflammatory response in adipose tissue of rhesus monkeys on a high-fat, high-sugar diet. Cell Metab. 18, 1–23. 10.1016/j.cmet.2013.09.004 24093677 PMC3832130

[B27] Jönsson C. Batista A. P. C. Kjølhede P. Strålfors P. (2019). Insulin and β-adrenergic receptors mediate lipolytic and anti-lipolytic signalling that is not altered by type 2 diabetes in human adipocytes. Biochem. J. 476, 2883–2908. 10.1042/BCJ20190594 31519735 PMC6792037

[B28] Juan M. E. Maijó M. Planas J. M. (2010). Quantification of trans-resveratrol and its metabolites in rat plasma and tissues by HPLC. J. Pharm. Biomed. Anal. 51, 391–398. 10.1016/j.jpba.2009.03.026 19406597

[B29] Ko K. Woo J. Bae J. Y. Roh H. T. Lee Y. H. Shin K. O. (2018). Exercise training improves intramuscular triglyceride lipolysis sensitivity in high-fat diet induced obese mice. Lipids Health Dis. 17, 1–7. 10.1186/s12944-018-0730-8 29661202 PMC5902881

[B30] Lagouge M. Argmann C. Gerhart-Hines Z. Meziane H. Lerin C. Daussin F.. (2006). Resveratrol improves mitochondrial function and protects against metabolic disease by activating SIRT1 and PGC-1alpha. Cell 127, 1109–1122. 10.1016/j.cell.2006.11.013 17112576

[B31] Lassailly G. Caiazzo R. Buob D. Pigeyre M. Verkindt H. Labreuche J.. (2015). Bariatric surgery reduces features of nonalcoholic steatohepatitis in morbidly obese patients. Gastroenterology 149, 379–388. 10.1053/j.gastro.2015.04.014 25917783

[B32] Lim R. Beekley A. Johnson D. C. Davis K. A. (2018). Early and late complications of bariatric operation. Trauma Surg. Acute Care Open 3, 1–7. 10.1136/tsaco-2018-000219 30402562 PMC6203132

[B33] Liu J. M. H. Zhang J. Zhang X. Hlavaty K. A. Ricci C. F. Leonard J. N.. (2016). Transforming growth factor-beta 1 delivery from microporous scaffolds decreases inflammation post-implant and enhances function of transplanted islets. Biomaterials 80, 11–19. 10.1016/j.biomaterials.2015.11.065 26701143 PMC4706476

[B34] Macarulla M. T. Alberdi G. Gómez S. Tueros I. Bald C. Rodríguez V. M.. (2009). Effects of different doses of resveratrol on body fat and serum parameters in rats fed a hypercaloric diet. J. Physiol. Biochem. 65, 369–376. 10.1007/BF03185932 20358350

[B35] Malandrino M. I. Fucho R. Weber M. Calderon-Dominguez M. Mir J. F. Valcarcel L.. (2015). Enhanced fatty acid oxidation in adipocytes and macrophages reduces lipid-induced triglyceride accumulation and inflammation. Am. J. Physiol. Endocrinol. Metab. 308, E756–E769. 10.1152/ajpendo.00362.2014 25714670

[B36] Mayerson A. B. Hundal R. S. Dufour S. Lebon V. Befroy D. Cline G. W.. (2002). The effects of rosiglitazone on insulin sensitivity, lipolysis, and hepatic and skeletal muscle triglyceride content in patients with type 2 diabetes. Diabetes 51, 797–802. 10.2337/diabetes.51.3.797 11872682 PMC2995527

[B37] Mercader J. Palou A. Bonet M. L. (2011). Resveratrol enhances fatty acid oxidation capacity and reduces resistin and Retinol-Binding Protein 4 expression in white adipocytes. J. Nutr. Biochem. 22, 828–834. 10.1016/j.jnutbio.2010.07.007 21109418

[B38] Mir J. F. Serra D. Mera P. Herrero L. Malandrino M. I. (2012). Mitochondrial Fatty Acid Oxidation in Obesity. Antioxid. Redox Signal. 19, 269–284. 10.1089/ars.2012.4875 22900819 PMC3691913

[B39] Montgomery M. K. Brown S. H. J. Mitchell T. W. Coster A. C. F. Cooney G. J. Turner N. (2017). Association of muscle lipidomic profile with high-fat diet-induced insulin resistance across five mouse strains. Sci. Rep. 7, 1–9. 10.1038/s41598-017-14214-1 29066734 PMC5654831

[B40] Murphy K. P. Hendley M. A. Isely C. Annamalai P. Peña E. Gower R. M. (2018). Resveratrol delivery from porous poly(lactide-co-glycolide) scaffolds promotes an anti-inflammatory environment within visceral adipose tissue. ACS Appl. Mater. Interfaces 10, 43363–43374. 10.1021/acsami.8b13421 30462474 PMC7076954

[B41] Nabais T. Brouillet F. Kyriacos S. Mroueh M. Amores da Silva P. Bataille B.. (2007). High-amylose carboxymethyl starch matrices for oral sustained drug-release: In vitro and in vivo evaluation. Eur. J. Pharm. Biopharm. 65, 371–378. 10.1016/j.ejpb.2006.12.001 17275270

[B42] O'Neill L. A. J. Kishton R. J. Rathmell J. (2016). A guide to immunometabolism for immunologists. Nat. Rev. Immunol. 16, 553–565. 10.1038/nri.2016.70 27396447 PMC5001910

[B43] Petersen K. F. Oral E. A. Dufour S. Befroy D. Ariyan C. Yu C.. (2002). Leptin reverses insulin resistance and hepatic steatosis in patients with severe lipodystrophy. J. Clin. Invest. 109, 1345–1350. 10.1172/JCI0215001 12021250 PMC150981

[B44] Petersen M. C. Shulman G. I. (2018). Mechanisms of insulin action and insulin resistance. Physiol. Rev. 98, 2133–2223. 10.1152/physrev.00063.2017 30067154 PMC6170977

[B45] Remmerie A. Scott C. L. (2018). Macrophages and lipid metabolism. Cell. Immunol. 330, 27–42. 10.1016/j.cellimm.2018.01.020 29429624 PMC6108423

[B46] Rutkowski J. M. Stern J. H. Scherer P. E. (2015). The cell biology of fat expansion. J. Cell Biol. 208, 501–512. 10.1083/jcb.201409063 25733711 PMC4347644

[B47] Sako K. Sawada T. Nakashima H. Yokohama S. Sonobe T. (2002). Influence of water soluble fillers in hydroxypropylmethylcellulose matrices on in vitro and in vivo drug release. J. Control. Release 81, 165–172. 10.1016/S0168-3659(02)00067-6 11992689

[B48] Schweiger M. Romauch M. Schreiber R. Grabner G. F. Hütter S. Kotzbeck P. (2017). Pharmacological inhibition of adipose triglyceride lipase corrects high-fat diet-induced insulin resistance and hepatosteatosis in mice. Nat. Commun. 8:14859 10.1038/ncomms14859 28327588 PMC5364409

[B49] Sheikh Z. Brooks P. J. Barzilay O. Fine N. Glogauer M. (2015). Macrophages, foreign body giant cells and their response to implantable biomaterials. Materials 8, 5671–5701. 10.3390/ma8095269 28793529 PMC5512621

[B50] Shin H. S. Kang S.-Il, Park, D.-B. Kim S. J. (2016). Resveratrol suppresses inflammatory responses and improves glucose uptake in adipocytes interacted with macrophages. Genes Genomics 38, 137–143. 10.1007/s13258-015-0348-4

[B51] Shulman G. I. (2014). Ectopic fat in insulin resistance, dyslipidemia, and cardiometabolic disease. N. Engl. J. Med. 371, 1131–1141. 10.1056/NEJMra1011035 25229917

[B52] Snel M. Jonker J. T. Schoones J. Lamb H. De Roos A. Pijl H.. (2012). Ectopic fat and insulin resistance: pathophysiology and effect of diet and lifestyle interventions. Int. J. Endocrinol. 2012:983814. 10.1155/2012/983814 22675355 PMC3366269

[B53] Spalding K. L. Bernard S. Näslund E. Salehpour M. Possnert G. Appelsved L.. (2017). Impact of fat mass and distribution on lipid turnover in human adipose tissue. Nat. Commun. 8:15253. 10.1038/ncomms15253 28534500 PMC5457499

[B54] Timmers S. Konings E. Bilet L. Houtkooper R. H. van de Weijer T. Goossens G. H.. (2011). Calorie restriction-like effects of 30 days of Resveratrol (resVida ™) supplementation on energy metabolism and metabolic profile in obese humans. Cell Metab. 14, 612–622. 10.1016/j.cmet.2011.10.002 22055504 PMC3880862

[B55] Turner N. Kowalski G. M. Leslie S. J. Risis S. Yang C. Lee-Young R. S.. (2013). Distinct patterns of tissue-specific lipid accumulation during the induction of insulin resistance in mice by high-fat feeding. Diabetologia 56, 1638–1648. 10.1007/s00125-013-2913-1 23620060

[B56] Williams L. M. Campbell F. M. Drew J. E. Koch C. Hoggard N. Rees W. D.. (2014). The development of diet-induced obesity and glucose intolerance in C57BL/6 mice on a high-fat diet consists of distinct phases. PLoS ONE 9:e106159. 10.1371/journal.pone.0106159 25170916 PMC4149520

[B57] Zolnik B. S. Burgess D. J. (2008). Evaluation of *in vivo-in vitro* release of dexamethasone from PLGA microspheres. J. Control. Release 127, 137–145. 10.1016/j.jconrel.2008.01.004 18282629

